# Stability assessment of housekeeping genes for qRT-PCR in *Yersinia enterocolitica* cultured at 22°C and 37°C

**DOI:** 10.1128/spectrum.01146-24

**Published:** 2024-10-04

**Authors:** Chuchu Li, Lu Zhou, Xiaoxuan Ma, Liguo Zhu, Jia Li, Lingning Meng, Mei Han, Danwei Wang, Han Shen, Chang Liu

**Affiliations:** 1 Department of Laboratory Medicine, Nanjing Drum Tower Hospital, Nanjing, Jiangsu, China; 2 Department of Acute Infectious Disease Control and Prevention, Jiangsu Provincial Center for Disease Control and Prevention, Nanjing, Jiangsu, China; City of Hope Department of Pathology, Duarte, California, USA

**Keywords:** *Yersinia enterocolitica*, housekeeping gene, qRT-PCR

## Abstract

*Yersinia enterocolitica*, a species within the genus *Yersinia*, thrives optimally at 22–25°C but can also grow at the mammalian core body temperature of 37°C. This dual temperature adaptability necessitates establishing both temperature conditions in research to examine the effects on various biological processes. In quantitative real-time PCR (qRT-PCR) assays, the selection of appropriate housekeeping genes is vital for data accuracy. Nevertheless, the lack of alternatives and information often leads to the default use of the 16S rRNA gene despite potential limitations. This investigation sourced 16 potential reference genes through a comprehensive review of the literature and transcriptome sequencing data analysis. We validated the expression stability of these genes via qRT-PCR across 12 *Y. enterocolitica* strains, representing the four prevalent serotypes O:3, O:5,27, O:8, and O:9, isolated from diarrheal patient stool samples. This approach aimed to minimize the impact of serotype heterogeneity. After acquiring Cq values, gene stability was evaluated using four established algorithms—ΔCq, geNorm, NormFinder, and BestKeeper—and subsequently synthesized into a consolidated ranking through the Robust Rank Aggregation (RRA) method. Our study suggests that the genes *glnS*, *nuoB*, *glmS*, *gyrB*, *dnaK*, and *thrS* maintain consistent expression across varying culture temperatures, supporting their candidacy as robust housekeeping genes. We advise against the exclusive use of 16S rRNA for this purpose. Should tradition prevail in its utilization, it must be employed with discernment, preferably alongside one or two of the housekeeping genes identified in this study as internal controls.

**IMPORTANCE:**

In our study, we focused on identifying stable reference genes for quantitative real-time PCR (qRT-PCR) experiments on *Y. enterocolitica* cultured at different temperatures (22°C and 37°C). After thoroughly evaluating 16 candidate genes, we identified six genes—*glnS*, *nuoB*, *glmS*, *gyrB*, *dnaK*, and *thrS*—as exhibiting stable expression across these temperature conditions, making them ideal reference genes for *Y. enterocolitica* studies. This discovery is crucial for ensuring the accuracy and reliability of qRT-PCR data, as the choice of appropriate reference genes is key to normalizing expression data and minimizing experimental variability. Importantly, our research extended beyond bioinformatics analysis by incorporating validation with clinical strains, bridging the gap between theoretical predictions and practical application. This approach not only underscores the robustness and reliability of our findings but also directly addresses the critical need for experimental validation in the field. By providing a set of validated, stably expressed reference genes, our work offers valuable guidance for designing experiments involving *Y. enterocolitica*, enhancing the reliability of research outcomes, and advancing our understanding of this significant pathogen.

## INTRODUCTION

*Yersinia enterocolitica* is a human pathogen belonging to the *Yersiniaceae* family, which is widely found in both the environment and animal reservoirs. It is a zoonotic bacterium that spreads through the fecal-oral route, generally leading to mild self-limiting enterocolitis, terminal ileitis or adenitis, and occasionally followed by chronic inflammatory diseases such as arthritis and erythema nodosum. *Y. enterocolitica* can adapt to various temperature ranges, but specific temperatures can influence its behavior and characteristics. Its optimal growth temperature is 22°C, at which it is a motile single cell with high lipopolysaccharide (LPS), O-polysaccharide (OPS) expression, and the highest growth rate. When transferred to a mammalian host or when the incubation temperature is raised to 37°C, it switches to a non-motile and autoagglutinating form and increases the expression level of virulence proteins such as invasin (Inv), *Yersinia* adhesin A (YadA), and plasmid-encoded *Yersinia* exoproteins (Yops). Therefore, culture temperature is an important aspect to consider when studying *Y. enterocolitica*, as well as *Y. pestis* and *Y. pseudotuberculosis*. In most studies of these bacteria, researchers typically divide their experiments into two groups based on temperature conditions: room temperature (approximately 22–25°C) and host temperature (37°C). Subsequent experiments are then conducted to observe and analyze the results obtained. This approach allows for a comprehensive understanding of the bacterial behavior and responses under different temperature environments.

Quantitative RT-PCR (qRT-PCR) is a popular technique for the quantification and comparative analysis of gene expression levels, valued for its rapidity, precision, and cost-efficiency. Housekeeping genes are indispensable internal controls in this methodology, used to normalize gene expression data and reduce experimental variation. Ideally, genes that exhibit stable expression throughout the experimental conditions should be selected as housekeeping genes. However, the process of choosing housekeeping genes often lacks careful consideration. Researchers frequently rely on housekeeping genes reported in previous literature, with 16s rRNA being the most commonly selected. The appropriateness of this conventional approach merits careful evaluation.

In this study, we leverage the differential gene expression characteristics of *Y. enterocolitica* at 22°C and 37°C as a starting point, aiming to carefully evaluate the stability of six “traditional housekeeping genes” including 16 s rRNA, that have been reported in existing literature, as well as ten novel “candidate housekeeping genes” identified from RNA-seq data downloaded from the Sequence Read Archive (SRA) database (https://www.ncbi.nlm.nih.gov/sra). The experimental validation was conducted using 12 *Y. enterocolitica* strains isolated from patients with diarrhea, with the strains’ serotypes encompassing as comprehensively as possible the most common serotypes of this bacterium, namely, O:3; O:5,27; O:8; and O:9, to eliminate heterogeneity between different serotypes.

## MATERIALS AND METHODS

### Sample collection

*Y.enterocolitica* strains were collected from the Chinese Pathogen Identification Net (China PIN) project (1) (Table 1). Each strain was isolated from the fecal specimens of a separate patient suffering from diarrhea. Organism identification was performed using API 20 E (bioMérieux, France) . The O-serogroup of the isolates was determined by slide agglutination test (Denka Seiken, Japan). The strains were initially stored in a −80℃ ultra-low temperature freezer. For revival, strains were cultured on columbia blood agar plates (OXOID) at 28°C. After successful revival, the strains were then cultured at both 22°C and 37°C for 24 h for subsequent qRT-PCR experiments.

**TABLE 1:** Strains used in this study

Sample ID	API code	Serotype	Biotype	Collection date
O:3-1	1155723	O:3	1A	2016
O:3-2	1014723	O:3	2	2018
O:3-3	1015523	O:3	3	2023
O:5-1	1155723	O:5,27	1A	2017
O:5-2	1155723	O:5,27	1A	2018
O:5-3	1155723	O:5,27	1A	2019
O:8-1	1155723	O:8	1A	2016
O:8-2	1155723	O:8	1A	2018
O:8-3	1055723	O:8	1A	2019
O:9-1	1055723	O:9	1A	2016
O:9--	1155723	O:9	1A	2016
O:9–3	1155723	O:9	1A	2020

### RNA-Seq data sets and transcript abundance analysis

*Y. enterocolitica* RNA-Seq data sets [BioProject: PRJNA264525 (2) and BioProject: PRJNA277186 (3, 4)], uploaded by a research team from the University of Helsinki, were obtained from the Sequence Read Archive (SRA) database. Both studies used *Y. enterocolitica* Y11 (serotype: O3) as the wild-type strain, and all strains were incubated at two temperatures, 22°C and 37°C, respectively, before sequencing. To improve the robustness of the screening outcomes, RNA-Seq data from genetically engineered mutant strains in these studies were also incorporated.

Raw FASTQ-files were extracted using the prefetch and fasterq-dump tool from the SRA Toolkit 3.02, low-quality reads, and adapters were trimmed using fastp 0.23.2 with default settings (5). *Y.enterocolitica* Y11 (RefSeq: GCF_000253175.1) genome was first annotated by Bakta (v1.8.1) and then used to build the transcriptome index. Transcript abundances were quantified using Kallisto v0.48.0 to get transcripts per million (TPM) values (6) (Table S1).

### Identification of novel candidate housekeeping genes from transcriptomes

Candidate reference genes that were stably expressed between 22°C and 37°C were identified using the method provided by Eisenberg and Levanon, with minor modifications (7). Briefly, Transcripts Per Kilobase Million was used instead of Reads Per Kilobase Million to represent gene expression levels, and the following four principles were used in the screening process of candidate housekeeping genes: (i) low variance between samples cultured at 22/37°C: standard deviation (SD) of log2 (TPM) <0.5; (ii) no exceptional expression in any single sample: Max |log_2_(TPM) – mean [log_2_(TPM)]| <1; (iii) relatively high expression levels: Mean [log_2_(TPM)] > 5; and (iv) highly conserved in the *Y. enterocolitica* genome. The R code used for the analysis can be found in File S2. To assess gene conservation, genomic data of *Y. enterocolitica* were obtained from the NCBI using the ncbi-genome-download tool (v0.3.1), and the conservation of candidate housekeeping genes was subsequently assessed by blastn (v2.12.0) comparison, with the filtering condition of having a %identity >95 match in at least 90% of the genome. Genes that met all of the above criteria were ranked in descending order of CV value, and the top 10 genes were selected for qRT-PCR validation. The scope of target genes was limited to coding sequences and ribosomal RNA (rRNA), with non-coding RNAs, small open reading frames, transfer RNAs, and transfer-messenger RNAs being specifically excluded from the study.

### Validation of the expression stability of target genes

The expression stability of housekeeping genes reported from previous literature (6 in total) as well as the candidate housekeeping genes screened in this study (10 in total) was verified by qRT-PCR (Table 2). Primers were designed using the Primer3Plus website tool (8). Total RNA was extracted using the Spin Column Bacteria Total RNA Purification Kit (Sangon, B518655) and the RNase-Free DNase Set (Sangon, B618253). RNA concentration was measured using the NanoDrop Lite Spectrophotometer (Thermo), and RNA purity was assessed by the ratio of OD260/280. qRT-PCR was conducted in a 96-well plate using the HiScript II One Step qRT-PCR SYBR Green Kit (Vazyme) and the BioRad CFX Compact, each reaction of 20 µL contained 10 µL 2 × One Step SYBR Green Mix, 1 µL One-Step SYBR Green Enzyme Mix, 0.4 µL each of 10 µM primers, and total of 1 ng RNA. Reactions were run using the cycling parameters of 50°C for 3 min to generate cDNA, 95°C for 30 s, 40 cycles of 95°C for 10 s, and 60°C for 30 s, followed by a single cycle at 95°C for 15 s, 60°C for 1 min, and 95°C for 15 s for melt curve analysis. Each qRT-PCR analysis was performed in triplicate, and melting curves were analyzed to assess whether the assays have produced single, specific products.

**TABLE 2:** List of primers used for qRT-PCR analysis

Gene^*a*^	Product	RefSeq	Primer sequence (5′-3′)	Product length
*nuoB*	NADH-quinone oxidoreductase subunit B	WP_005159213.1	GGGTTATTTCCATGGGTGCC ATATTGGCGCGGTAAACACC	226
*gyrB*	DNA topoisomerase (ATP-hydrolyzing) subunit B	WP_005161097.1	GAAGTGGCATTGCAGTGGAA CTTTCACGGAAACCACAGCA	223
*dnaK*	Molecular chaperone DnaK	WP_005157035.1	GGTATGGATCTGCGTACCGA CAGTGACTCCAACTTAGCGC	180
*rrs*	16S ribosomal RNA	N/A^*b*^	ATACCCTGGTAGTCCACGCT AGTTGCAGACTCCAATCCGG	236
*gmk*	Guanylate kinase	WP_005159213.1	AGTGGGGCAGGGAAATCAAG CGCCTTGCCAGTCGATATCT	286
*rpoB*	DNA-directed RNA polymerase subunit beta	WP_005165796.1	TCAAAGAGTGCCAGATCCGT ACGATGCAGCTGAGATACGA	209
*rpoD*	RNA polymerase sigma factor RpoD	WP_005160832.1	TCTGGTCAACAACATGCGTG TGCACATCGTCCGAAACATC	210
*thrS*	Threonine—tRNA ligase	WP_005161572.1	GAAAACCGCGAGTACTGCAT CATCCTGAGTAAAGCCACGC	181
*ftsY*	Signal recognition particle-docking protein FtsY	WP_016266354.1	TGTGGAAGAGTCTCAGGCTG CCCATAAAACCGGAGCCAAG	186
*rlmL*	Bifunctional 23S rRNA methyltransferase RlmK/RlmL	WP_005160003.1	CGGGTGAATGTTTTCCTGCA ACCTGAACCACACATCGGAT	192
*rpoN*	RNA polymerase factor sigma-54	WP_005162476.1	ATCTCACTGTGCCACTGGAA CTAATGACCAGGCGTGCTTC	205
*dnaX*	DNA polymerase III subunit gamma/tau	WP_005158173.1	GCGTTAGTGAGTGCAGATGG CTGGATATCTGTAGGCGGCA	216
*glmS*	Glutamine—fructose-6-phosphate transaminase (isomerizing)	WP_005161161.1	GTCGTCTTGAATACCGTGGC GTATGTGCAATCCCGGTTCC	154
*lepA*	Translation elongation factor 4	WP_005159409.1	GCTCCGATGACTATGAGGCT GTGCCGTCGTGATAAGTTCC	195
*argS*	Arginine—tRNA ligase	WP_133964390.1	TGATCGTCAATGGGTTGCCA GATGGTCGAGCGCAAATGAC	151
*glnS*	Glutamine—tRNA ligase	WP_005163363.1	CCGTCGCTTGTATGATTGGG CGAATTGATGCTGCGGTGTA	213

^*a*^ The underlined genes are the six housekeeping genes previously used in *Yersinia* sp. studies, and the remaining 10 are candidate housekeeping genes screened from the transcriptome sequencing data in this study.

^*b*^ The 16S ribosomal RNA encoded by the rrs gene is not a protein. Therefore, it is indicated as N/A in the RefSeq column.

The stability of the expression of the selected genes was assessed using RefFinder, a web-based tool for evaluating the stability and reliability of reference genes, by integrating the results of major computational programs including geNorm (9), NormFinder (10), BestKeeper (11), and the comparative delta-Ct method (12). The comprehensive ranking was conducted using the Robust Rank Aggregation (RRA) package in R (13).

## RESULTS

### Selection of novel candidate housekeeping genes based on transcriptome sequencing data

**Fig 1:**
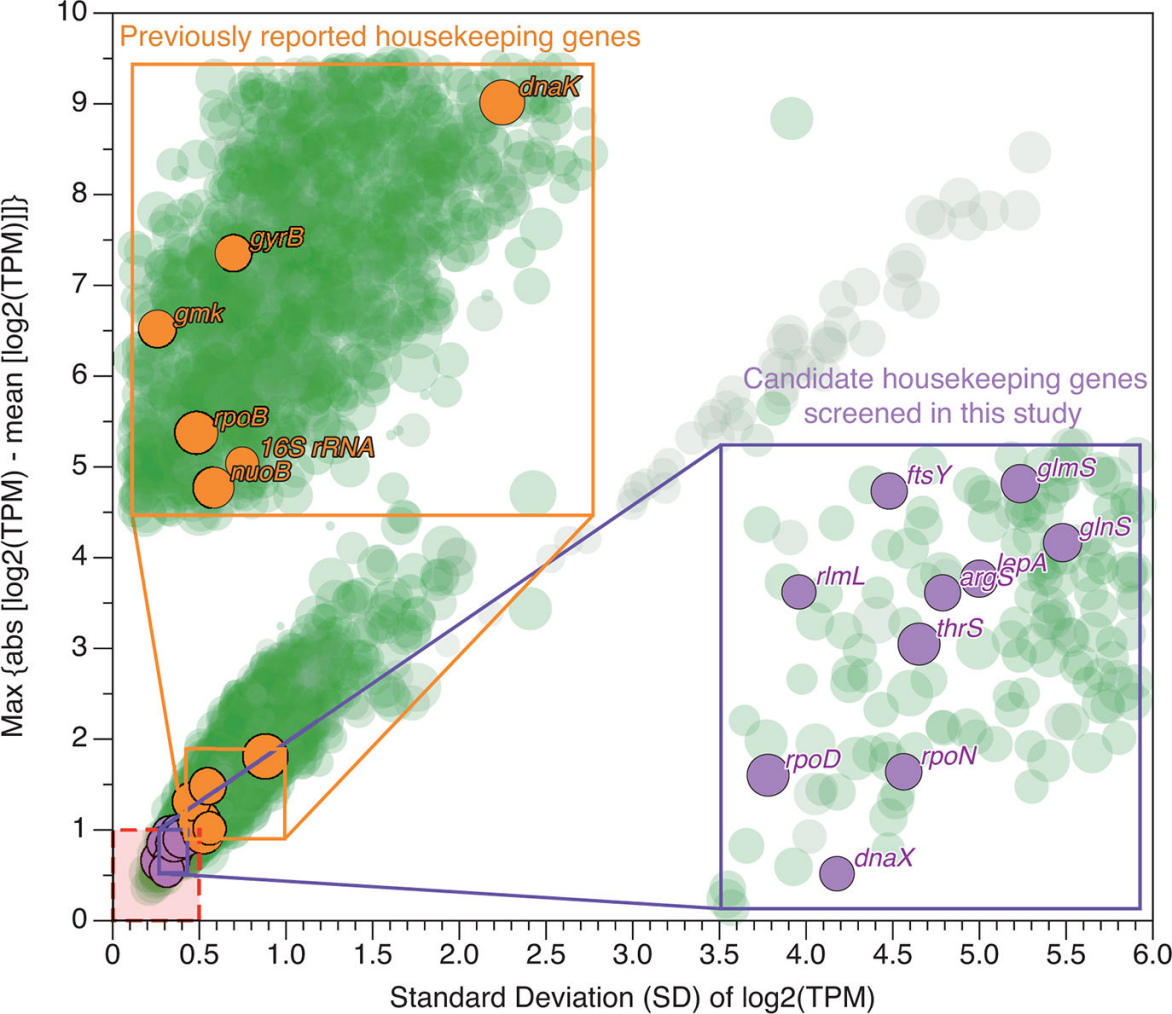
Scatter plot of log2(TPM) gene expression levels across 22°C and 37°C. The size of the circles mirrors the gene expression levels, with larger circles denoting higher expression levels. The color gradient signifies the level of conservation, with greener hues indicating superior conservation, otherwise tending toward gray. The area highlighted in red represents the range of candidate reference genes examined in this study.

**Fig 2:**
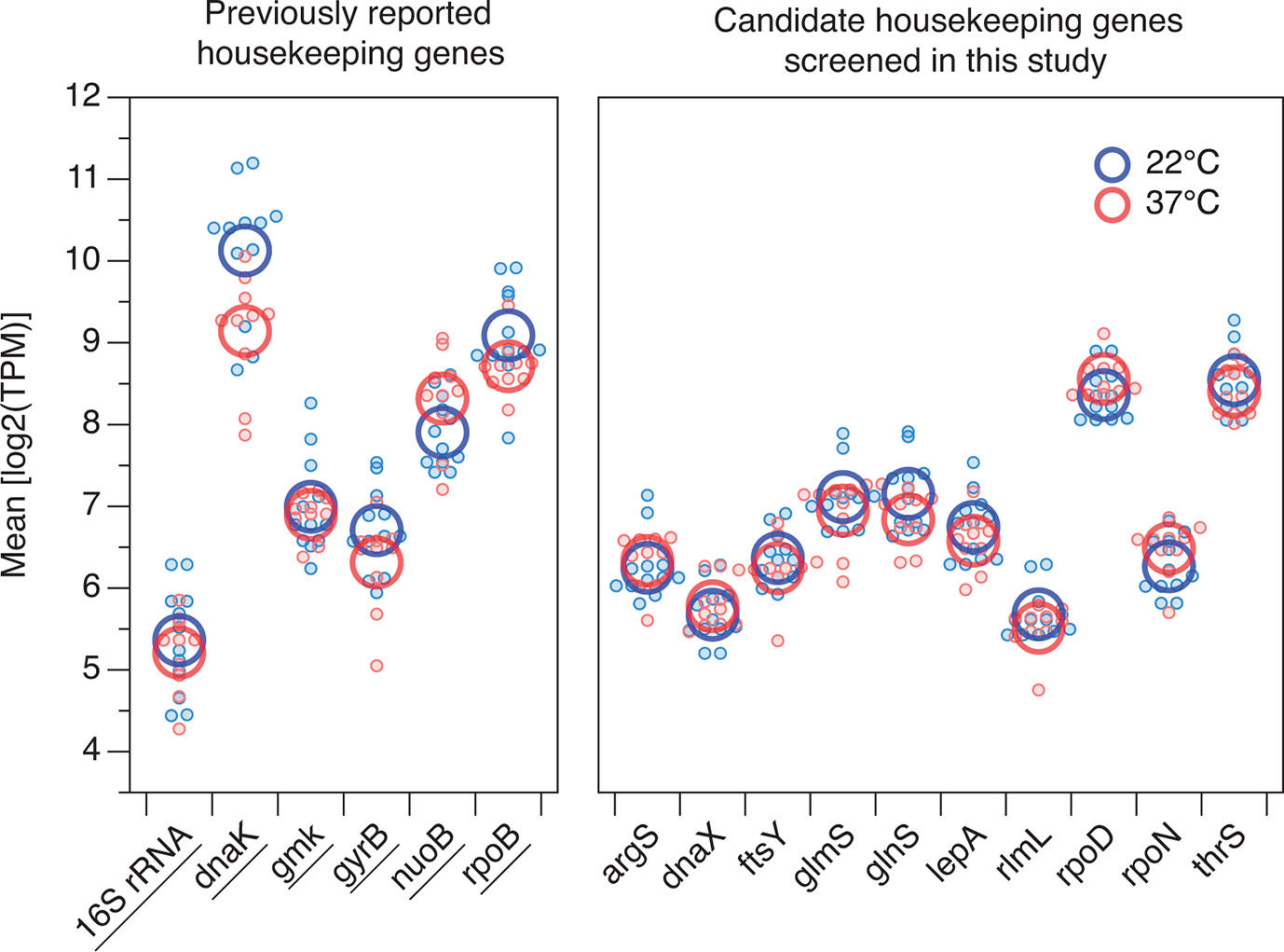
Gene expression difference plot of the target gene at 22°C and 37°C. The gene expression levels of the target gene at 22°C and 37°C are represented by blue and red circles, respectively. The small circles indicate the values of individual strains, while the large circles indicate the average values of the small circles. Six previously reported housekeeping genes are separated into a group, and their gene names are underscored.

By analyzing RNA-Seq data, we acquired TPM values for all genes in *Y. enterocolitica* Y11. We computed both the log2(TPM) values and the maximum absolute deviation for each gene’s log2(TPM). These values were then plotted in a scatter plot (Fig. 1). In this plot, genes positioned closer to the bottom-left corner indicate minimal expression variability due to temperature and inter-strain differences. To identify novel candidate housekeeping genes, we delineated a stringent criterion (marked by the red box in Fig. 1) and selected 10 genes that exhibited sufficient sequence conservation, relatively high expression levels, and the lowest coefficient of variation (CV) for their log2(TPM) values. The genes identified were *rpoD*, *thrS*, *ftsY*, *rlmL*, *rpoN*, *dnaX*, *glmS*, *lepA*, *argS*, and *glnS*. To provide a clear visual comparison of the expression stability at 22°C and 37°C for the six housekeeping genes previously reported in the literature and the 10 newly identified candidate housekeeping genes, we constructed Fig. 2. The proximity between the red and blue circles represents the stability of gene expression across the two temperatures: the closer the circles, the more stable the gene expression at 22°C and 37°C.

### Acquiring Cq values of housekeeping genes in clinical strains cultured at 22°C/37°C

The subsequent step in our research was to simulate a realistic laboratory scenario by validating the expression stability of 16 genes identified in previous work at temperatures of 22°C and 37°C, using *Y. enterocolitica* strains. The primer sequences for these experiments are detailed in Table 2. These primers have been confirmed to effectively amplify the target genes, and their melting curves exhibit a single peak, indicating the specificity of amplification (data not shown). To lend more credibility to our experimental outcomes, we refrained from utilizing standard strains and instead employed clinical samples isolated from the feces of patients with diarrhea. Moreover, to reduce the impact of heterogeneity among different serotypes, we made a concerted effort to collect a comprehensive set of strains from four serotypes: O:3, O5:27, O:8, and O:9, securing three strains from each serotype. This resulted in a total of 12 bacterial strains for qRT-PCR validation.

**Fig 3:**
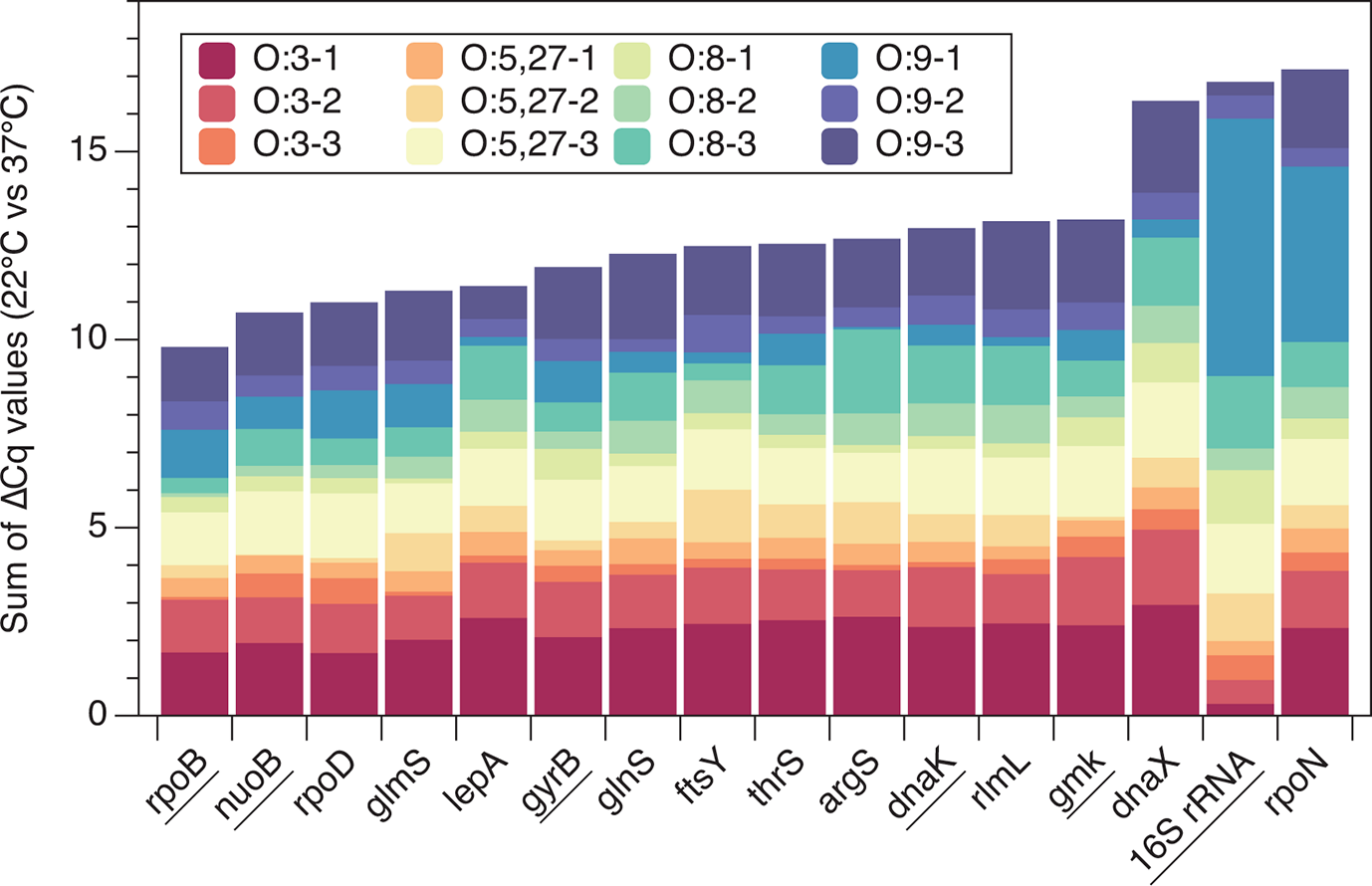
Cumulative bar chart of candidate housekeeping gene ∆Cq values across 22°C−37°C. The absolute values of the differences between the 22°C and 37°C Cq values for each strain were calculated and summed. Six housekeeping genes previously reported in the literature are underscored.

After obtaining the Cq values for each strain and gene, we initially computed the sum of the ΔCq values at 22°C and 37°C for each gene (Fig. 3). It is tentatively concluded that genes with smaller sums of ΔCq values are more stable in their expression levels. When ranking the sum of ΔCq values for each gene in ascending order, the four genes (top 25%) with the smallest values were *rpoB*, *nuoB*, *rpoD*, and *glmS*. However, when evaluating the stability within each of the four serotypes separately, a total of nine unique genes emerged. Among these, *nuoB, rpoB*, and *rpoD* were identified three times, *glmS* twice, and *argS, dnaK*, *gyrB*, *lepA*, and 16 s rRNA each appeared once.

### Analysis of gene expression stability by different statistical algorithms

Next, the expression stability of the 16 target candidate housekeeping genes was assessed by three dedicated analytical tools: *geNorm* (9), *NormFinder* (10), and *BestKeeper* (11) algorithms.

**Fig 4:**
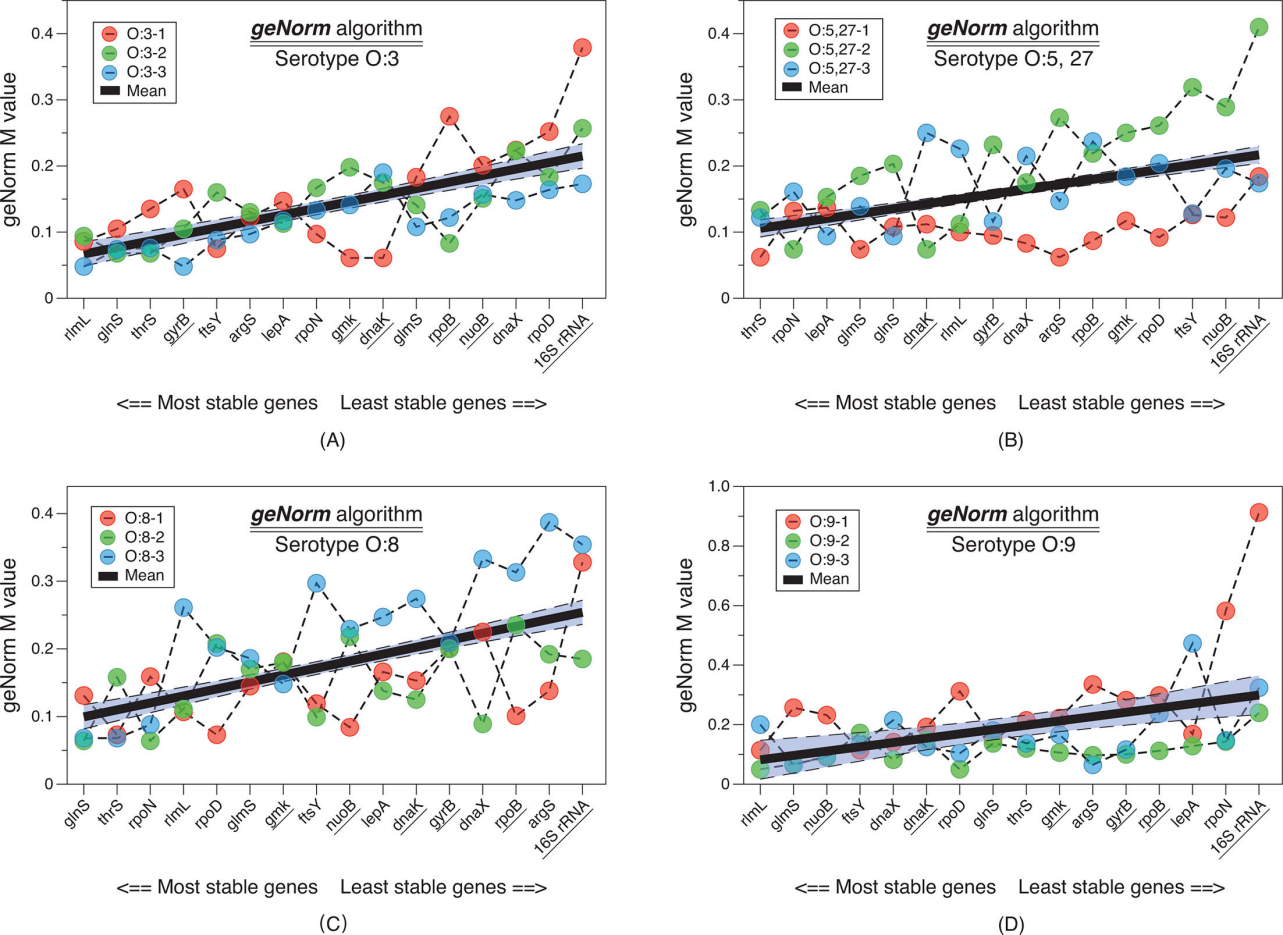
Line graph of *M*-values calculated by the geNorm algorithm for evaluating gene stability. The strains were categorized into four groups based on serotypes: O:3, O:5,27, O:8, and O:9. The individual values of three strains within each group were plotted, and the average values for each gene were determined and sorted in ascending order based on their averages. Smaller values indicate more stable expression levels. Six housekeeping genes previously reported in the literature are underscored.

*GeNorm* is the most commonly used algorithm for assessing the stability of candidate housekeeping genes. It calculates a gene-stability measure value (M) by evaluating the average pairwise variation of each candidate gene in comparison to other genes. A lower M value indicates greater stability. After calculating M values for all genes, the two genes with the lowest scores are recommended as housekeeping genes. The experimental bacterial strains were categorized into four groups based on their serotypes. The *M* values for each gene within the three strains in each group were computed and arranged in ascending order according to their average values (Fig. 4). The top 25% genes with the smallest *M* values from each serotype group (four genes per group) were selected, resulting in a total of nine unique genes. Among them, *glnS*, *rlmL*, and *thrS* appeared three times each, *rpoN* appeared twice, and the remaining five genes *ftsY, glmS, gyrB*, *lepA,* and *nuoB* appeared only once.

**Fig 5:**
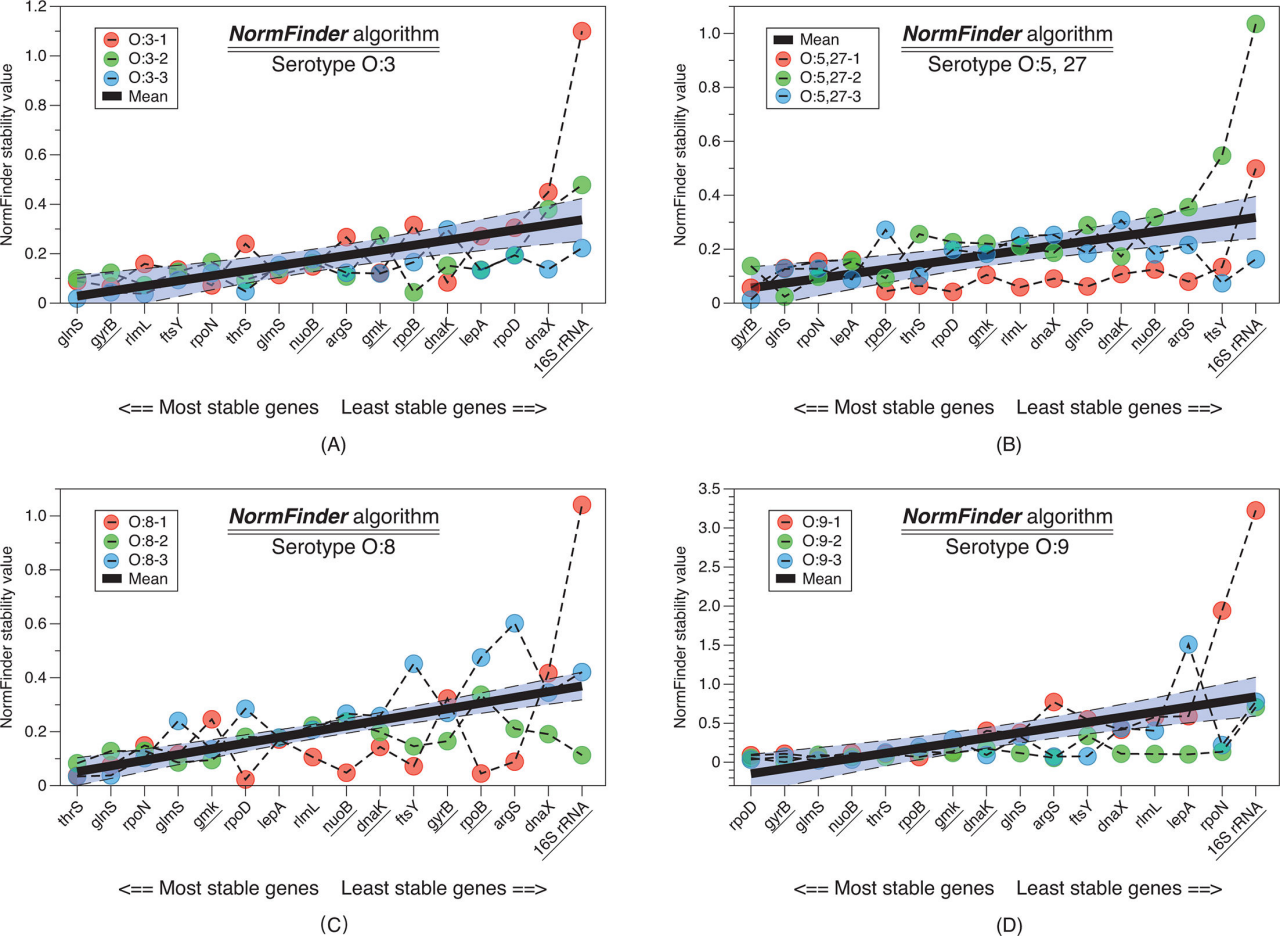
Gene stability indices calculated using the NormFinder algorithm. The data were grouped into four categories based on the serotypes of the bacteria: O:3, O:5,27, O:8, and O:9, with the stability indices for each gene within each group of three strains plotted individually. After calculating the average stability index for each gene, they were sorted in ascending order based on their averages. Smaller values indicate more stable expression levels. Six housekeeping genes previously reported in the literature are underscored.

*NormFinder* is another popular algorithm for evaluating gene expression stability. It adopts a model-based approach to assess stability and calculates a stability value for each gene by considering both the overall variation and the variation attributed to specific sample groups. Similarly, a lower score from this algorithm indicates higher stability of the gene (Fig. 5). The top 25% of genes with the smallest *NormFinder* scores were selected within each serotype group, resulting in 16 genes, out of which 10 were unique. Among them, *glnS* and *gyrB* appeared three times, *glmS* and *rpoN* appeared twice, while the remaining six genes *ftsY*, *lepA*, *nuoB*, *rlmL*, *rpoD,* and *thrS* appeared once.

**Fig 6:**
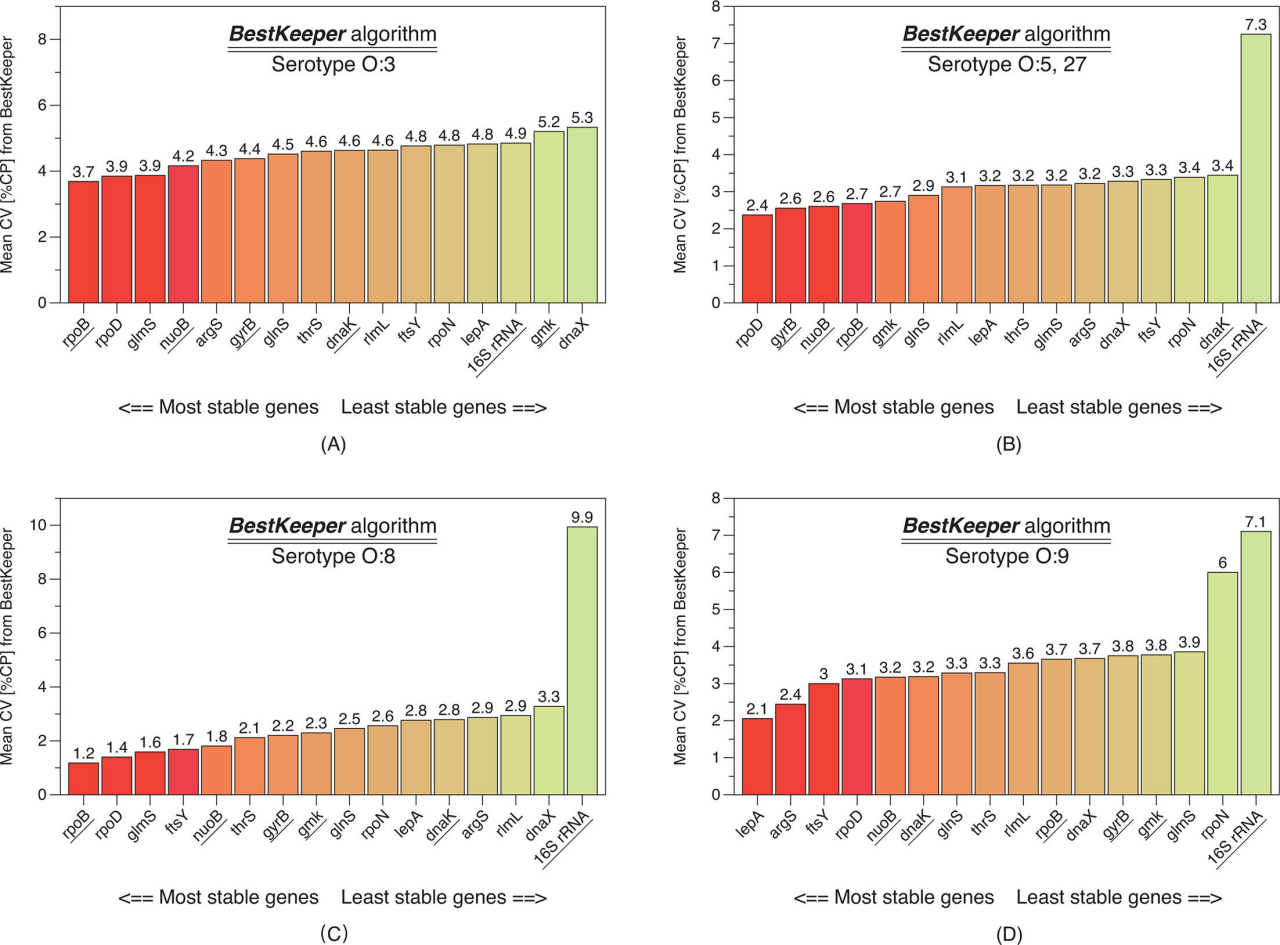
CV values calculated using the BestKeeper algorithm. The data are grouped into four categories based on the serotypes of the experimental strains: O:3, O:5,27, O:8, and O:9. After calculating the average CV value for each gene across the strains, the genes are sorted in ascending order. Genes with lower rankings indicate better stability in gene expression. Six housekeeping genes previously reported in the literature are underscored.

The *BestKeeper* algorithm calculates the stability of candidate genes by analyzing their standard deviation (SD) of Cq values, as well as the coefficient of variance (CV), correlation coefficient (*r*), and *P*-value (*P*), which are also important parameters (Fig. 6). It separately calculates the top 25% most stable genes in each serotype group, resulting in a total of 16 genes, out of which 8 are unique. Among them, *rpoD* is counted four times, *ropB* is counted three times, *ftsY*, *glmS*, and *nuoB* are each counted two times, and *argS*, *gyrB*, and *lepA* are each counted one time.

### Comprehensive ranking of housekeeping gene stability using the robust rank aggregation algorithm

**Fig 7:**
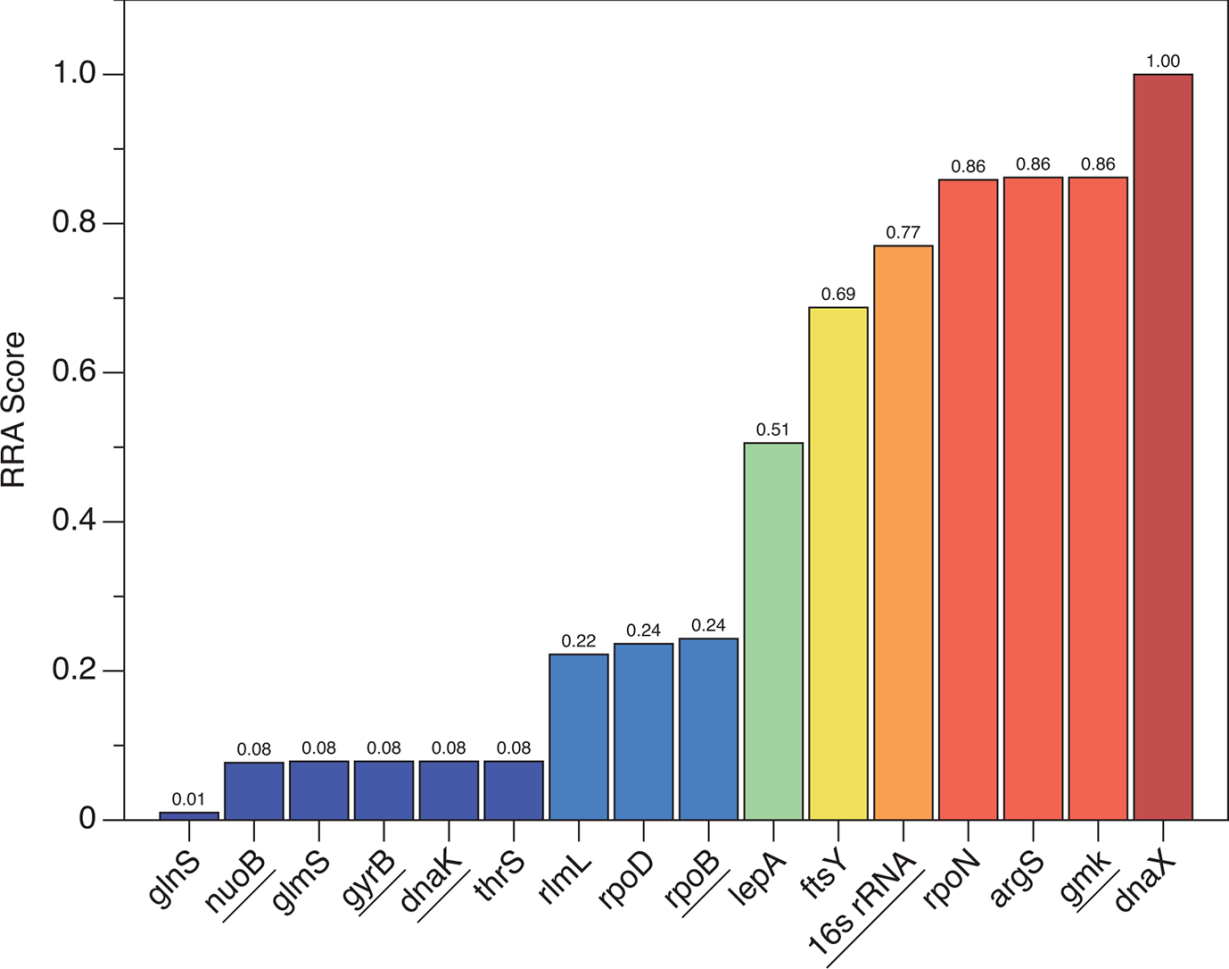
Integration of candidate housekeeping gene stability rankings using the RRA algorithm. The RRA algorithm was utilized to merge rankings derived from the ∆Cq, GeNorm, NormFinder, and BestKeeper methods into an overall ranking. A lower RRA score indicates that the gene consistently ranks higher across multiple lists, reflecting a stable expression profile; conversely, a higher RRA score is indicative of variable rankings across different lists or uniformly lower positions. In this study, genes that ranked higher (marked in blue) demonstrated better stability overall compared to those that ranked lower (marked in red).

It is evident that even for the same set of qRT-PCR data, different algorithms may yield rankings that are not entirely consistent. To obtain a composite ranking, we employed the robust rank aggregation (RRA) algorithm to integrate 48 ranking results from 12 clinical bacterial strains obtained through 4 different evaluation methods. This method led us to a comprehensive ranking (Fig. 7). The results show that the composite rankings of the 16 candidate housekeeping genes can generally be divided into three tiers. The first tier includes *glnS*, *nuoB*, *glmS*, *gyrB*, *dnaK*, and *thrS*, which have the lowest RRA scores, indicating their consistently high rankings and good stability across various algorithms. *glnS* stands out in particular, with an RRA score of only 0.01, placing it in the foremost position. The second-tier genes, *rlmL*, *rpoD*, and *rpoB*, display moderate overall ranking performance. The third-tier comprises *lepA*, *ftsY*, *16s rRNA*, *rpoN*, *argS*, *gmk*, and *dnaX*, which suggests that their rankings fluctuate significantly across different algorithms or they tend to rank lower more consistently.

## DISCUSSION

In recent years, an increasing number of studies have begun to employ transcriptomic and even proteomic approaches to observe differences in bacterial protein expression. However, qRT-PCR, as a traditional and mature technology, remains one of the most commonly used and reliable techniques in microbial laboratories. This is due to its low technical threshold, widespread availability of equipment, low reagent costs, good reproducibility, and particularly because its results are intuitive and easy to analyze.

When conducting relative quantification experiments using this technique, it is necessary to designate a “housekeeping gene” as an “internal control” and use it as a “baseline” to assess changes in the expression levels of other genes. The selection of an appropriate housekeeping gene has always been a topic of interest. In mammalian studies, commonly used housekeeping genes include GAPDH (Glyceraldehyde 3-phosphate dehydrogenase), ACTB (β-actin), and B2M (β-2 microglobulin), which encode either cytoskeletal proteins or are involved in basic biological functions and are expressed relatively stably and consistently across various cell types. However, further research has revealed that the expression levels of these commonly used housekeeping genes can fluctuate in different organs or under specific types of external disturbances (14). Therefore, a consensus has been reached that housekeeping genes should be carefully evaluated and selected according to specific experimental conditions to minimize the generation of experimental errors as much as possible (7).

In microbial research, although some studies have assessed the stability of housekeeping genes in different types of bacteria under various environmental conditions and offered selection recommendations, researchers often rely on consulting published literature when choosing housekeeping genes. Particularly, 16S rRNA is often the first choice as a housekeeping gene in most qRT-PCR experiments due to its presence and relative conservation in all bacteria, as well as its prominence in microbial classification and identification. Although in some conditions, 16S rRNA can fulfill the role of a housekeeping gene (15, 16), in reality, no single gene, including 16S rRNA, can achieve absolute stability under all conditions. After careful evaluation based on experimental conditions, more scientific choices often emerge (17–20).

To effectively adapt to the internal environments of humans and other mammals, the optimal growth temperature for most human pathogens is 37°C. However, *Yersinia*, a genus of bacteria that includes psychrophilic organisms, can grow at temperatures ranging from 0°C to 4°C, with an optimal range of 22–28°C. Despite this, at 37°C, the body temperature of humans and other mammals, these bacteria can still effectively infect and proliferate (21). Since temperature changes significantly affect various physiological characteristics of the bacterium, including virulence, morphology, phage cleavage, lipopolysaccharide structure, researchers often set both 22–25°C and 37°C as cultivation temperatures. This allows them to observe and understand the effects of temperature changes on their scientific questions of interest (22–24). However, little research has focused on how the expression levels of housekeeping genes change at different temperatures.

Based on the background mentioned above, this study aims to assess the stability of housekeeping genes in *Y. enterocolitica* at two cultivation temperatures, 22°C and 37°C. We identified a list of genes for testing through both existing literature and transcriptome sequencing data analysis, collected 12 clinical strains covering the four most common serotypes for qRT-PCR validation, and after obtaining Cq values, assessed them using four classic algorithms, integrating the assessment results to provide a comprehensive ranking. Our results show that *glnS*, *nuoB*, *glmS*, *gyrB*, *dnaK*, and *thrS* exhibit minor differences in expression levels when the cultivation temperature changes, and we recommend them as housekeeping genes. We advise against solely designating 16s rRNA as a housekeeping gene. However, if it is still preferred, it should be treated with caution, and one or two of the housekeeping genes recommended in this study should be selected to serve as the “internal reference” for the research.

### Conclusions

Our study indicates that simply selecting housekeeping genes based on reference literature is inappropriate in bacterial research. The use of 16S rRNA as a housekeeping gene requires careful consideration. In Yersinia enterocolitica, for its most common experimental variable—the two cultivation temperatures of 22°C / 37°C, the expression levels of *glnS*, *nuoB*, *glmS*, *gyrB*, *dnaK*, and *thrS* show minor differences, and we recommend designating one or more of them as housekeeping genes in qRT-PCR experiments.

## Data Availability

The TPM values are provided in Table S1. The R script used in this study is available in the Supplemental data.

## SUPPLEMENTAL MATERIAL

Supplemental data spectrum.01146-24-s0001.txt R code used.

Table S1 spectrum.01146-24-s0002.docx TPM values calculated from fastq files.

ASM does not own the copyrights to Supplemental Material that may be linked to, or accessed through, an article. The authors have granted ASM a non-exclusive, world-wide license to publish the Supplemental Material files. Please contact the corresponding author directly for reuse.
