# Supplementary material for: Stability assessment of housekeeping genes for qRT-PCR in Yersinia enterocolitica cultured at 22°C and 37°C
Source: Microbiol Spectr. 2024 Oct 4;12(11):e01146-24. doi: 10.1128/spectrum.01146-24 (PMC11536982; doi:10.1128/spectrum.01146-24)
Supplement: Table S1 — TPM values calculated from fastq files. [file spectrum.01146-24-s0002.docx]

| **Table 1.** Strains used in this study | | | | |
| --- | --- | --- | --- | --- |
| Sample ID | API Code | Serotype | Biotype | Collection Date |
| O:3-1 | 1155723 | O:3 | 1A | 2016 |
| O:3-2 | 1014723 | O:3 | 2 | 2018 |
| O:3-3 | 1015523 | O:3 | 3 | 2023 |
| O:5-1 | 1155723 | O:5,27 | 1A | 2017 |
| O:5-2 | 1155723 | O:5,27 | 1A | 2018 |
| O:5-3 | 1155723 | O:5,27 | 1A | 2019 |
| O:8-1 | 1155723 | O:8 | 1A | 2016 |
| O:8-2 | 1155723 | O:8 | 1A | 2018 |
| O:8-3 | 1055723 | O:8 | 1A | 2019 |
| O:9-1 | 1055723 | O:9 | 1A | 2016 |
| O:9-2 | 1155723 | O:9 | 1A | 2016 |
| O:9-3 | 1155723 | O:9 | 1A | 2020 |
